# Lumbosacral transitional vertebra causing Bertolotti’s syndrome: a case report and review of the literature

**DOI:** 10.4076/1757-1626-2-8320

**Published:** 2009-07-06

**Authors:** Georgios Paraskevas, Alexandros Tzaveas, Georgios Koutras, Konstantinos Natsis

**Affiliations:** Orthopaedic Department, Panagia HospitalNik. Plastira 22, N. Krini, 55132, Kalamaria, ThessalonikiGreece

## Abstract

**Introduction:**

Lumbosacral transitional vertebra is an anatomical variation of the fifth lumbar vertebra in which an enlarged transverse process can form a joint or fusion with the sacrum or ilium. The association of that variant with low back pain and the change in the biomechanical properties of the lumbar spine is called Bertolotti’s syndrome.

**Case presentation:**

We report a case of a 40-year-old male patient with chronic low back pain extending to the left buttock, just above the ipsilateral sacroiliac joint. Radiographic investigation revealed an anomalous enlargement of the left transverse process of the fifth lumbar vertebra forming a pseudarthrosis with the infrajacent ala of the sacrum.

**Conclusion:**

In young patients with back pain the possibility of Bertolotti’s syndrome should always be taken in account.

## Introduction

“Bertolotti’s syndrome” is characterised by the presence of a variation of the fifth lumbar vertebra having a large transverse process, either articulated or fused with the sacral basis or iliac crest, and producing a chronic, persistent low back pain [1,2]. Bertolotti stated as early as in 1917 that these abnormal vertebrae may produce low back pain due to arthritic changes occurring at the site of pseudarthrosis [3]. That not rare anatomic variant is reported as having an incidence of 4% to 21% [2,4]. Recently, a very high incidence of 30% has been reported [5].

Whether such an anatomical variation produces or not low back pain and/or sciatica is a subject of great debate. Some authors believe that the lumbosacral transitional vertebra could cause symptoms of back pain and/or sciatica [1,4,6,7], while others claim that this abnormal vertebra does not affect their incidence [5,8,9].

## Case presentation

A 40-year-old male presented at the outpatient department for further evaluation of his left low back pain. He was of Caucasian origin, non-smoker, with no relevant family or medical history. His height was 1.70 m and weight 85 kg. He was on paracetamol for the last 3 weeks. His symptoms began years before presentation after performing certain movements, without any history of trauma, and mainly while bending forward on strenuous exercise. Initially, he described a sensation of pulled muscles in his right left back region which was activity-related. The pain was not as excruciating as to prevent him from undertaking normal activities and patient had no difficulty in walking neither being awakened up by the pain during the night.

Physical examination demonstrated tenderness over the lumbar spine and the area of the left sacrum, provoked by superficial and deep palpation. Laseque sign was negative bilaterally. Reflexes, sensation and muscle power were normal on both lower limbs.

**Figure 1.**
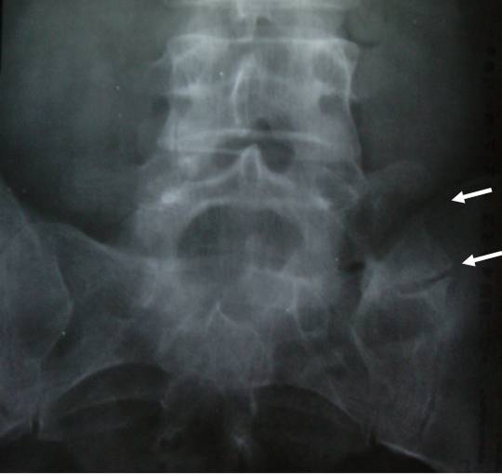
Anteroposterior radiograph of the lumbar spine showing enlargement of the left transverse process of the fifth lumbar vertebra. Upper arrow: enlarged transverse process; lower arrow: anomalous articulation with the sacral ala with arthritic changes.

The radiographs demonstrated a typical lumbosacral transitional vertebra (Figure 1), with an extremely large left transverse process of the fifth lumbar vertebra, articulating with the ala of sacrum. In addition, the plain radiograph revealed degenerative changes of the pseudarthrosis. Injection with local anaesthetic and steroid led to pain alleviation. Patient reassured and conservative treatment was offered, including analgesics, non steroid anti-inflammatory drugs, physiotherapy and exercises program. He was instructed to come for follow up upon worsening of the symptoms or appearance of any neurological deficit.

## Discussion

According to Castellvi’s classification, there are four types of lumbosacral transitional vertebrae, type I, dysplastic transverse process with height > 90 mm, type II, incomplete lumbarisation/sacralisation, type III, complete lumbarisation/sacralisation with complete fusion with the neighboring sacral basis and type IV, mixed [2].

There are various reports regarding the histopathological and radiological changes appearing at the adjacent structures of the lumbosacral transitional vertebra. It has been suggested that the lumbosacral transitional vertebra decreases the annulus fibrosis degeneration of the disc below, without having the same effect on endplates and nuclear complex [5]. An association has been found between lumbosacral transitional vertebra and disc herniations as well as facet joint degeneration [2,4]. Otani et al stated that a lumbosacral transitional vertebra was found more often in patients with disc herniation (17%) than in the control group (11%) [10]. It has been demonstrated that the discs immediately above the transitional vertebra were significantly more degenerative (disc protrusion or extrusion) compared with the disc found between the transitional vertebra and the sacrum [4,11]. Also, nerve root canal stenosis has been found at the level suprajacent to the transitional vertebra [4]. It has been reported that there is no difference in the prevalence of spondylolysis or spondylolisthesis between patients with transitional vertebra and group controls [12], and that there is no relationship between lumbosacral transitional vertebra and a congenitally narrower canal [4]. It has been also suggested that the lumbosacral transitional vertebra causes degenerative changes on the opposite facet joint [7].

Luoma et al hypothesized that because of the restriction of rotational and bending movements by the pseudarthroses the L5-S1 disc is protected from traumatic events [5]. According to Castellvi et al the transitional vertebrae cause abnormal torque movements above these anomalous vertebrae, a fact that could result in disc degeneration [2]. Aihara et al in an anatomical study of 70 cadavers claimed that the iliolumbar ligament at the level immediately above the transitional vertebra is much thinner and weaker than in cadavers without a lumbosacral transitional vertebra. Especially the posterior bands of the ligament at this level have the appearance of fascia rather than of a ligament. Due to that condition disc degeneration may occur at higher vertebral levels more frequently than level L5-S1. The same authors found the iliolumbar ligaments at the lumbosacral transitional vertebra consisting of dense fibrous connective tissue, thus protecting the L5-S1 disc [11].

There is so much controversy with regards to whether such an abnormal vertebra produces or not symptoms of low back pain. Many authors supported a relationship between lumbosacral transitional vertebra and low back pain [5,8,9], while Wigh et al [13] and Castellvi et al [2] found that in patients with back pain and sciatica, the transitional vertebra had a prevalence of 21% and 30% respectively. The type II transitional vertebra in which a diarthrosis exists between transverse process and sacrum has been said to be related to low back pain [14]. In the same type an increased number of disc prolapses have been reported at the level above the transitional vertebra [2]. It has been reported that facetogenic low back pain could arise from a contralateral transitional vertebra of type II [7]. Our case is classified as type II lumbosacral transitional vertebra, as an asymmetric pseudarthrosis with arthritic changes is formed.

Otani et al supported that the transitional vertebra does not influence the incidence of nerve root symptoms. However, they claimed that this vertebra in patients with disc herniation or lumbar canal stenosis without spondylolisthesis may be a risk factor for the development of nerve root symptoms [10]. Quinlan et al found the total incidence of Bertolotti’s syndrome being 4.6%, while the frequency was 11.4% in under 30-year age group. These authors claimed that the transitional vertebra should be kept in mind when low back pain is appeared in young individuals [6]. Vergauwen et al demonstrated that the abnormal vertebra does not constitute a risk factor for spine degenerative changes, but when degeneration occurs it is focused on the suprajacent level of the transitional vertebra [4]. In our case we consider that the localized pain was caused by the degenerative changes of the anomalous articulation between the transverse process of the transitional vertebra and the ala sacrum.

With direct local injection of anaesthetic and steroid within the cavity of pseudarthrosis successful resolution of low backache has been reported [15]. Brault et al performed corticosteroid and local anaesthetic injection to the contralateral facet joint to the enlarged transverse process of the transitional vertebra [7]. Santavirta et al recommended that when the conservative treatment fails and no disc pathology occurs then resection of the abnormal transverse process may be tried. The same authors suggested posterolateral fusion if the transitional disc appears to be pathological and the suprajacent disc remains intact [1].

## Conclusion

The list of differential diagnosis should always include Bertolotti’s syndrome, when investigating back pain in young patients. The treatment, whether conservative or operative, is still debated. In our case we performed injection with local anesthetic and steroid within the abnormal articulation and after a follow-up of 18 months patient reported an improvement of his symptoms, however not complete resolution.
